# Advances in Research on Bacterial Oxidation of Mn(II): A Visualized Bibliometric Analysis Based on CiteSpace

**DOI:** 10.3390/microorganisms12081611

**Published:** 2024-08-07

**Authors:** Wentao Mo, Hang Wang, Jianghan Wang, Yue Wang, Yunfei Liu, Yi Luo, Minghui He, Shuang Cheng, Huiting Mei, Jin He, Jianmei Su

**Affiliations:** 1 Hubei Key Laboratory of Regional Development and Environmental Response, Faculty of Resources and Environmental Science, Hubei University, Wuhan 430062, China; mowentao0513@163.com (W.M.); wanghang23101220@jhun.edu.cn (H.W.); 202321108012145@stu.hubu.edu.cn (J.W.); 18566199997@163.com (Y.W.); 202021108011313@stu.hubu.edu.cn (Y.L.); luoyi031622@163.com (Y.L.); 18380305308@163.com (M.H.); 202221108012295@stu.hubu.edu.cn (S.C.); 202221108012247@stu.hubu.edu.cn (H.M.); 2 National Key Laboratory of Agricultural Microbiology, College of Life Science and Technology, Huazhong Agricultural University, Wuhan 430062, China; hejin@mail.hzau.edu.cn

**Keywords:** Mn(II) oxidation, manganese oxides, Mn(II)-oxidizing bacteria, CiteSpace, bibliometric analysis, visualized knowledge map

## Abstract

Manganese (Mn) pollution poses a serious threat to the health of animals, plants, and humans. The microbial-mediated Mn(II) removal method has received widespread attention because of its rapid growth, high efficiency, and economy. Mn(II)-oxidizing bacteria can oxidize toxic soluble Mn(II) into non-toxic Mn(III/IV) oxides, which can further participate in the transformation of other heavy metals and organic pollutants, playing a crucial role in environmental remediation. This study aims to conduct a bibliometric analysis of research papers on bacterial Mn(II) oxidation using CiteSpace, and to explore the research hotspots and developmental trends within this field between 2008 and 2023. A series of visualized knowledge map analyses were conducted with 469 screened SCI research papers regarding annual publication quantity, author groups and their countries and regions, journal categories, publishing institutions, and keywords. China, the USA, and Japan published the most significant number of research papers on the research of bacterial Mn(II) oxidation. Research hotspots of bacterial Mn(II) oxidation mainly focused on the species and distributions of Mn(II)-oxidizing bacteria, the influencing factors of Mn(II) oxidation, the mechanisms of Mn(II) oxidation, and their applications in environment. This bibliometric analysis provides a comprehensive visualized knowledge map to quickly understand the current advancements, research hotspots, and academic frontiers in bacterial Mn(II) oxidation.

## 1. Introduction

Manganese (Mn) is widely distributed in terrestrial and aquatic ecosystems globally, ranking as the second most abundant transition metal element in the Earth’s crust after iron [1]. Mn is also a crucial trace element in all living organisms, serving as a constituent or activator for numerous metalloenzyme [2]. However, due to the influence of natural processes or human activities, a substantial amount of Mn is released into the environment, leading to severe Mn pollution [3]. The Mn-polluted water and soil impact the growth of plants and crops and pose a severe threat to human health since they are transmitted through the food chain [4]. Therefore, addressing Mn(II) pollution problem has become one of the global environmental challenges [5].

Methods for the remediation of Mn(II) pollution encompass physical, chemical, and biological approaches [6]. Microbial remediation, one of the biological approaches, refers to the process in which microorganisms such as bacteria and fungi catalyze the oxidation of soluble Mn(II) to insoluble Mn(III/IV) oxide deposits through enzymatic and metabolic activities [7,8]. The microbial oxidation of Mn(II) is the leading cause of the environment’s formation of Mn(III/IV) oxides. Biological Mn oxides (BioMnO_x_) have a large specific surface area, multiple structural vacancies, and high reactivity, which can strongly adsorb and oxidize various heavy metals, nutrient elements, and organic matter, thereby affecting their concentration, morphology, chemical behavior, and bioavailability in the environment [9,10]. It has been found that Mn(II)-oxidizing microorganisms and the resultant BioMnO_x_ are involved in the oxidation and degradation processes of certain lignin compounds, humic substances, particulate organic carbon, organic pesticides, and antibiotics [11,12,13,14]. Therefore, Mn(II)-oxidizing microorganisms and the resulting BioMnO_x_ play crucial roles in the biogeochemical cycles of Mn and other elements, global ecological balance, climate change, and the environmental remediation of pollutants [15].

The currently identified Mn(II)-oxidizing bacteria include (1) Firmicutes: *Bacillus* [16,17] and *Brevibacillus* [18,19]; (2) Actinobacteria: *Agromyces* [20], *Microbacterium* [21] and *Cupriavidus* [22]; (3) α, β and γ Proteobacteria: *Pedomicrobium* [23], *Erythrobacter* [24], *Aurantimonas* [25], *Roseobacter* [26,27], *Leptothrix* [28,29,30,31], *Ralstonia* [32], *Pseudomonas* [33,34], *Citrobacter* [35], *Pantoea* [36], *Escherichia* [37,38], and many other genera. The most extensively studied Mn(II)-oxidizing bacteria are the model strains *Bacillus* sp. SG-1 [16], *Pseudomonas putida* MnB1 and GB-1 [39], and *Leptothrix discophora* SS-1 [29].

To date, there have been extensive studies on the isolation and identification of Mn(II)-oxidizing bacteria, as well as the characteristics of the formed BioMnO_x_. However, these bacteria exhibit significant differences in the catalytic mechanisms of Mn(II) oxidation. It is imperative to summarize the global research hotspots, scientific progress, and development trends quickly in the diverse field of Mn(II) oxidation by various bacteria, but there is no bibliometric analysis research in this field. In light of this, the present study aims to conduct a bibliometric analysis utilizing the CiteSpace software 6.3.R1 (64-bit), which can explore the key pathways and inflection points of knowledge within the field of discipline by mapping a series of visual charts [40]. This study analyzed research papers on bacterial Mn(II) oxidation published from the PubMed and Web of Science Core Collection database (WOSCC) over the past fifteen years (2008–2023), including publication years, countries and regions, authors, institutions, keywords, and other relevant aspects. This study systematically summarized the current advancements, tracked research focal points in the field of Mn(II)-oxidizing bacteria, and provided potential directions for the future promotion of bacterial Mn(II) oxidation research.

## 2. Materials and Methods

### 2.1. Data Source and Retrieval Strategy

**Figure 1:**
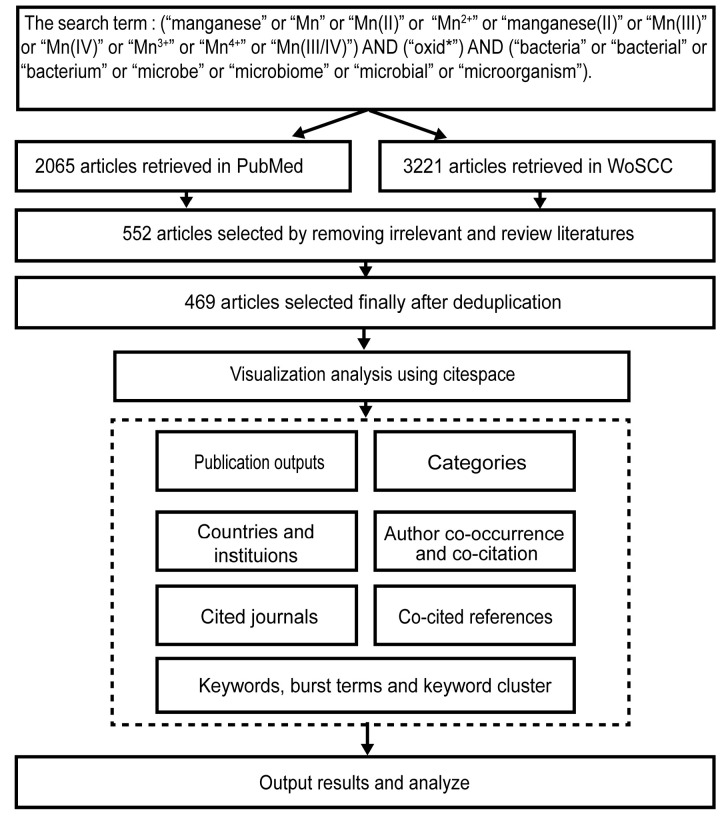
Literature retrieval and screening process in two databases.

Literature data were collected from PubMed (https://pubmed.ncbi.nlm.nih.gov) and WOSCC (https://www.webofscience.com/wos). The “Text Word” field was selected in the advanced search on PubMed. The following search terms were used for retrieval: “manganese” or “Mn” or “Mn(II)” or “Mn^2+^” or “manganese(II)” or “Mn(III)” or “Mn(IV)” or “Mn^3+^” or “Mn^4+^” or “Mn(III/IV)”, along with “oxid*”, and “bacteria” or “bacterial” or “bacterium” or “microbe” or “microbiome” or “microbial” or “microorganism”. The symbol “*” denotes a truncation symbol in the advanced search on PubMed, indicating a search for words prefixed with the preceding term. The inclusion criteria for the study were limited to the language (English) and document type (Article). The publication years were restricted to the period between 2008 and 2023. Subsequently, we conducted the exact search in the WOSCC using the “TS” (topic) field. The literature search was completed within one day to mitigate potential biases arising from database updates. Duplicate articles imported from both databases were removed using Zotero-6.0.36, and manual screening was performed to exclude reviews and articles not aligned with the thematic focus. The retrieval strategy is illustrated in Figure 1.

### 2.2. Data Analysis and Visualization

CiteSpace software 6.3.R1 (64-bit) is a scientific measurement visualization analysis software developed based on Java SE Development kit 21 (64-bit). This software offers a variety of visualization features, including the display of organizational structure distribution, author collaboration networks, comprehensive integration of research papers, keyword co-occurrence analysis, and keyword clustering. The visualization maps are primarily composed of numerous nodes and interwoven lines. The nodes represent different subject categories, and their sizes indicate the number or citation frequency. Node centrality measures the degree to which a node in the network occupies a central position in the paths connecting other nodes. Nodes with higher centrality positioned at the center of the network serve as transitional nodes for other peripheral nodes. If the periphery of a node appears in bright red or purple, it indicates that the node is a burst node or has a centrality greater than 0.10. Lines connecting two nodes signify relationships such as connection, collaboration, or co-citation [41,42]. In-depth analysis and summarization of the information represented by nodes and lines in the knowledge graph enable a quick understanding of emerging trends, research focal points, and developmental trends in the field. This study utilized CiteSpace software (version CiteSpace 6.3.R1, 64-bit) to conduct a bibliometric analysis on the field of bacterial Mn(II) oxidation, including disciplinary classification, countries and institutions, co-cited journals, authors and co-cited authors, keyword co-occurrence, and other aspects. The parameters for CiteSpace were set as follows: time range (from 2008 to 2023); term source (title, abstract, author keywords, weighted keywords); node types (successively selecting authors, institutions, countries, keywords, categories, co-cited references, cited authors, and cited journals); intensity calculation method (cosine similarity); and scope (within each slice). Subsequently, knowledge graph analysis and summarization were performed on selected research papers on bacterial Mn(II) oxidation from 2008 to 2023.

## 3. Results

### 3.1. Trend Analysis of Annual Publications

**Figure 2:**
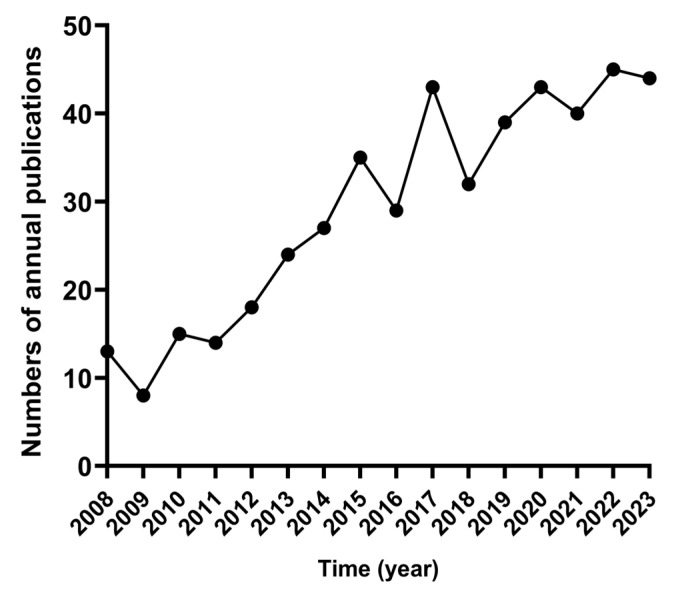
Numbers of annual publications from 2008 to 2023.

As shown in Figure 1, a total of 2065 research papers were retrieved from PubMed, and 3221 research papers were obtained from WOSCC. After the removal of reviews, duplicates, and irrelevant research papers, 469 research papers were ultimately chosen for subsequent visualization graph analysis. Figure 2 illustrates the number of research papers published in the field of bacterial Mn(II) oxidation from the year 2008 to 2023. The results indicate an overall increasing trend in the publication count of relevant research papers from 2008 to 2023. The numbers of research papers published in the years 2021, 2022, and 2023 were 40, 45, and 44, respectively. These data reflect that the study of bacterial Mn(II) oxidation remains a steadily growing hot research field on a global scale, constantly attracting the attention of more scientists and the participation of researchers. Moreover, with the continuous development of research techniques and the deepening of scientific research, more and more research papers on Mn(II) oxidation will be published.

### 3.2. Analysis of Countries and Institutions

**Figure 3:**
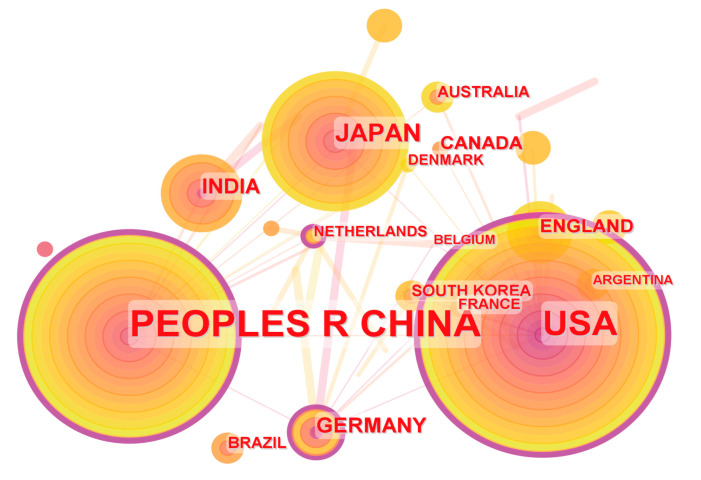
The countries of the authors and the collaborative networks among these countries.

Figure 3 illustrates the countries of these research papers’ authors and the collaboration network among authors from different countries, where the size of the circles represents the number of research publications from each country. Among the 469 research papers analyzed in this study, a total of 85 countries were involved. From Figure 3 and Table 1, it is observed that China is the most productive country, contributing a total of 197 research papers, followed by the USA (128 research papers), and Japan (33 research papers). The countries with centrality greater than 0.1 are the USA (0.38), China (0.34), Germany (0.15), and the Netherlands (0.14), indicating that these four countries had the highest proportion of international cooperation with other countries in the publications in this field. 

**Figure 4:**
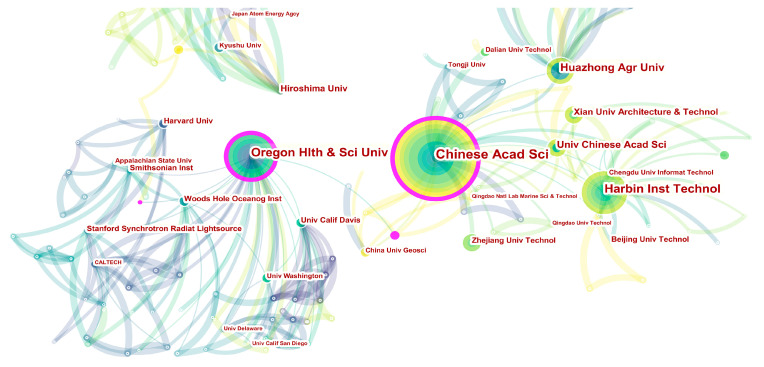
Collaboration network map of institutions.

These 469 research papers were published by 356 research institutions worldwide. As shown in Table 2, the institution with the most prolific research output is the Chinese Academy of Sciences (41), followed by Harbin Institute of Technology (28), Oregon Health and Science University (27), Huazhong Agricultural University (20), and University of Chinese Academy of Sciences (14), respectively. According to the publications, the top ten productive institutions are predominantly located in China and the USA, consistent with the distribution of countries. Figure 4 reveals the collaborative network relationships among these institutions. The overall collaborative network can be mainly divided into three sub-networks: China, the USA, and Japan. In the Chinese sub-network, the key collaborating institutions are the Chinese Academy of Sciences, Harbin Institute of Technology, and Huazhong Agricultural University, with a centrality of 0.19, 0.06, and 0.07, respectively. In the sub-networks of the USA or Japan, Oregon Health and Science University and Hiroshima University emerge as major collaborative nodes, with a centrality of 0.23 and 0.05, respectively. Evidently, institutional cooperation within the same country in this field is very close, while international cooperation is relatively rare, highlighting that geographical location may be a potential key factor influencing collaboration among authors.

### 3.3. Analysis of Disciplinary Classifications

**Figure 5:**
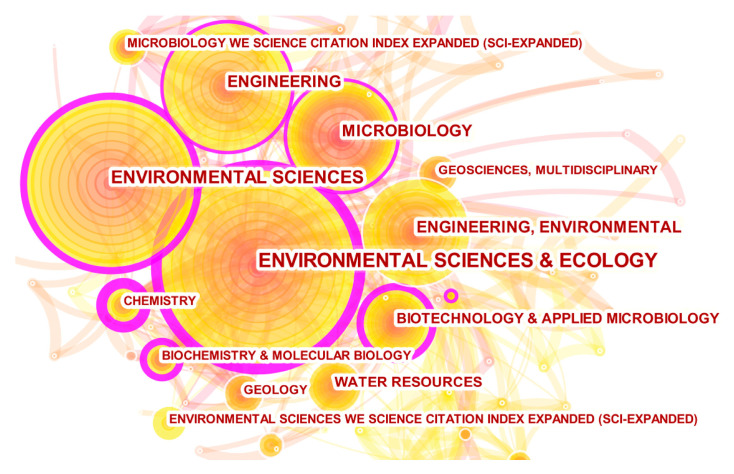
Co-occurrence network map of subject categories.

Through CiteSpace’s co-occurrence analysis of disciplinary classification, we gained in-depth insights into the research disciplines related to bacterial Mn(II) oxidation. The data reveal that these 469 research papers span 92 different disciplinary areas, indicating that the research topic of bacterial Mn(II) oxidation has received widespread attention from multiple disciplines. Table 3 lists the top ten disciplines by the frequency and centrality of publications. The results show that “Environmental Sciences and Ecology” (151; 0.41) has the highest research frequency and centrality among all disciplines, followed by “Environmental Sciences” (122; 0.32), “Engineering” (87; 0.18), “Microbiology” (73; 0.15), and “Biotechnology & Applied Microbiology” (52; 0.39). Additionally, the outer circles of these nodes in the co-occurrence network are all purple in Figure 5, indicating the significance of these disciplines in the study of bacterial Mn(II) oxidation. Considering both frequency and centrality, we can conclude that research on bacterial Mn(II) oxidation is primarily concentrated in the multi-disciplinary fields of “Environmental Science & Ecology”, “Chemistry”, “Biotechnology & Applied Microbiology”, and “Biochemistry & Molecular Biology”, suggesting that interdisciplinary theories and methods can be applied in the future to comprehensively study and reveal the mystery of the bacterial Mn(II) oxidation process.

### 3.4. Analysis of Authors of Co-Occurrence and Co-Citation

The analysis of author co-occurrence relationships can help understand their research output and country affiliation, and the collaboration patterns among authors. Co-cited author analysis can reflect the author’s influence in the field. There are 473 authors involved in these 469 research papers. Table 4 provides detailed information on the top ten authors with the highest number of published research papers and the top ten authors with the most co-citations. These ten authors collectively contribute to 25.34% of the total research output, with the majority coming from the USA and China, consistent with the earlier findings on national and institutional distribution. Bradley Tebo has the highest research output and has published 22 related papers in this field. Yaohui Bai published 13 research papers, while Jiuhui Qu and Jie Zhang each contributed 12 research papers. Notably, Bradley Tebo is also the author with the most citations (212), followed by Christopher Francis (104), Deric Learman (94), Mario Villalobos (89), Kati Geszvain (87), Gregory Dick (80), Samuel Webb (79), Naoyuki Miyata (74), Christopher Anderson (71), and Geert-Jan Brouwers (69).

**Figure 6:**
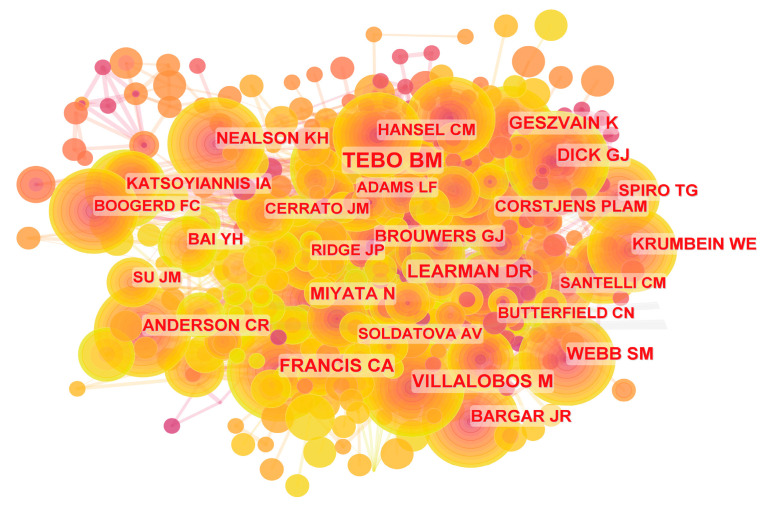
Network map of co-cited authors.

These authors also played a crucial bridging role within the co-cited author network (Figure 6). Among them, Bradley Tebo stands out as the largest node in the co-cited author network and acts as a central connector, indicating that he occupies a core position in this research field, and his research contributions receive significant attention and recognition. His research mainly focuses on two aspects: the isolation and identification of Mn(II)-oxidizing bacteria and the study of the molecular mechanisms of Mn(II) oxidation, revealing the distribution of Mn(II)-oxidizing bacteria in freshwater and marine environments, as well as their roles in Mn cycling and diverse ecosystems [43,44,45,46]. Therefore, future research in bacterial Mn(II) oxidation should actively track and continuously pay attention to the subsequent research outcomes of Bradley Tebo’s team.

### 3.5. Analysis of Journal Citations

Given that the impact of journals is primarily determined by citation frequency, a journal’s citation analysis reflects the distribution of foundational knowledge and crucial research outcomes within a specific field [47]. According to the analysis of journal co-citations, these 469 papers were cited from 433 journals. Table 5 outlines the top ten most frequently cited journals, along with their impact factors and respective countries.

The ten most cited journals are *Applied and Environmental Microbiology* (301 citations), *Environmental Science & Technology* (232 citations), *Water Research* (223 citations), *Geochimica et Cosmochimica Acta* (214 citations), *Geomicrobiology Journal* (205 citations), *Journal of Bacteriology* (188 citations), *Proceedings of the National Academy of Sciences of the United States of America* (180 citations), *Annual Review of Earth and Planetary Sciences* (168 citations), *Chemosphere* (158 citations), and *PLoS One* (153 citations). Among them, four journals have impact factors (IF) exceeding 10: *Annual Review of Earth and Planetary Sciences* (14.9), *Water Research* (12.8), *Proceedings of the National Academy of Sciences of the United States of America* (11.1), and *Environmental Science & Technology* (11.4). According to the Journal Citation Reports (JCRs) 2022–2023 standard, six of these journals are classified as high-quality Q1 journals. Eight of the top ten journals are based in the USA, and two are from England, indicating the USA provides a central and reliable platform for publishing relevant research papers.

### 3.6. Analysis of Co-Cited References

If one or more research papers are simultaneously cited by two or more subsequent research papers, this group of papers is referred to as co-cited references. These frequently co-cited references inevitably exhibit similarities in research content and related citation basis, which can be used to judge and screen research papers with significant impacts in the field. In this study, a total of 557 co-cited references were identified, represented by 557 nodes and 2391 links in the citation network graph. Table 6 lists the top ten research papers with the highest co-citation counts. These ten research papers were first published in 2011 and the latest in 2019. Two of the top ten co-cited references were authored by Kati Geszvain, who also ranked fifth in the co-cited author analysis in Table 4. From the topics of these ten research papers, most research papers have discovered different Mn(II)-oxidizing bacteria, such as *P. putida* MnB1, *P. putida* GB-1, *Roseobacter*, and *B. pumilus* WH4, which catalyze the Mn(II) oxidation with new mechanisms, such as the discovery of new Mn(II) oxidation factors, new multicopper oxidase genes, and heme peroxidase genes, or non-enzymatic catalysis processes mediated by superoxide production [39,48,49,50]. For instance, our research team purified the active multicopper oxidase CotA in *B. pumilus* WH4 through heterologous expression for the first time. It confirmed that the recombinant protein CotA could directly oxidize Mn(II) in vitro [51]. Additionally, some other research papers explored the practical applications of Mn(II)-oxidizing bacteria, such as their usage in biofilters to significantly enhance the removal capacity of heavy metals from water and degrade organic pollutants [52,53,54]. In summary, these most frequently co-cited references often reported important discoveries, broke through particular technological bottlenecks, or developed specific applications of Mn(II)-oxidizing bacteria, which were of great significance for bacterial Mn(II) oxidation research.

### 3.7. Analysis of Temporal and Burst of Keywords

Keywords represent highly refined and summarized research content, which serves as essential indicators reflecting research themes and hotspots. Table 7 lists the top 15 keywords with the highest frequency and centrality. Among them, the centrality of the six keywords “oxidation” (0.16), “iron” (0.19), “absorption” (0.13), “identification” (0.10), and “microbial community” (0.11) is more significant than 0.1, indicating that these core keywords play a crucial role as critical nodes in connecting other keywords.

**Figure 7:**
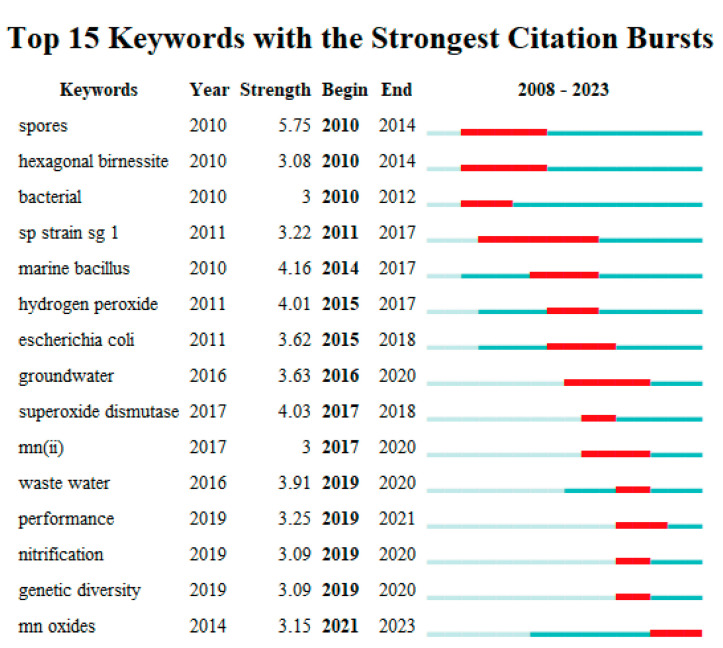
Top 15 keywords with the strongest citation bursts.

Burst keywords refer to suddenly appearing high-citation terms within a specific period. The strength and duration of these burst terms are essential indicators to infer the research frontier in a particular field. The periods when keywords appear are represented by red bars [58]. This study extracted 15 keywords with the most robust citation bursts based on their occurrence time. From Figure 7, it can be observed that from 2010 to 2017, the intensity and duration of keywords such as “spore”, “marine *bacillus*”, “hydrogen peroxide”, “biogenic Mn oxide”, “superoxide dismutase”, “binding”, “bacterial”, “identification”, and “*Escherichia coli*” are relatively significant. This indicates that researchers mainly focus on identifying Mn(II)-oxidizing bacteria types and exploring the mechanisms and influencing factors of bacterial Mn(II) oxidation. During this period, our research team cloned and expressed multicopper oxidase CotA and CueO from Mn(II)-oxidizing bacteria *B. pumilus* WH4 and *Escherichia coli*, respectively. We found the factors affecting their Mn(II) oxidation activity and the identification of generated biogenic Mn(III/IV) oxides [51,59,60]. However, superoxide dismutase cannot directly participate in Mn(II) oxidation and can promote Mn(II) absorption and heat production by recombinant strains [60].

From 2018 to 2023, burst keywords such as “bacterial”, “marine *bacillus*”, “groundwater”, “wastewater”, “performance”, “nitrification”, “genetic diversity”, and “Mn oxides” appeared. This indicated that researchers at this time were more concerned about applying Mn(II)-oxidizing bacteria and Mn(III/IV) oxides in practical environments. Based on the changes in the temporal distribution and bursts of these keywords, we concluded that the research concentrated in the field had shifted from the molecular theoretical studies of bacterial Mn(II) oxidation in the early stages towards the application research of Mn(II)-oxidizing bacteria and Mn(III/IV) minerals in the later stage.

### 3.8. Analysis of Keyword Clusters

**Figure 8:**
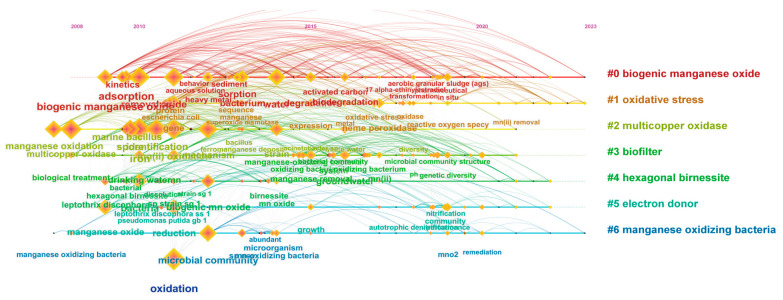
Keyword cluster.

Keyword clustering analysis involves classifying and concentrating numerous keywords to select the primary clustering labels and high-frequency keywords, and then presenting them as a visualized graph. S ≥ 0.70 indicates a convincing clustering result in the keyword clustering graph, while S ≥ 0.50 suggests a reasonable clustering. Q ≥ 0.30 signifies a significant community structure in the clustering. This study extracted 361 keywords from 469 research papers, resulting in 361 nodes and 2117 links (Figure 8). The S value is 0.77, and the Q value is 0.40, indicating the keyword clustering in this study is convincing.

Figure 8 displays the clustering results, categorizing keywords related to bacterial Mn(II) oxidation into seven classes: #0 biological manganese oxide (keywords: biogenic manganese oxides; aerobic granular sludge; adsorption; sediment; biodegradation; removal; transformation; kinetics; in situ; heavy metal; activated carbon; aqueous solution; behavior); #1 oxidative stress (keywords: oxidative stress; hydrogen peroxide; heme peroxidase; superoxide dismutase; gene; protein; *Escherichia coli*; reactive oxygen species); #2 multicopper oxidase (keywords: manganese oxidation; marine *bacillus*; spore; multicopper oxidase; iron; protein; identification; mechanism; diversity; expression); #3 biofilters (keywords: manganese removal; drinking water; microbial community structure; system; pH; genetic diversity; groundwater; manganese oxidizing bacteria; biological manganese oxidation; biological manganese oxides); #4 hexagonal birnessite (keywords: bacterial hexagonal birnessite; birnessite; *Leptothrix discophora* SS-1; strain SG-1; dissolution; mechanism; attraction; biological Mn oxides; optimal generation conditions; biogeochemical cycle); #5 electron donors (keywords: manganese oxide; reduction; growth; performance; *Leptothrix discophora* SS-1; *Pseudomonas putida* GB-1; nitrification; community; autotrophic denitrification; multicopper oxidation; particle attached bacteria); #6 manganese oxidizing bacteria (keywords: microbial community; abundant microorganism; remediation; MnO_2_; moving bed biofilm reactor; manganese oxides; toxicity; manganese removal; manganese oxidizing bacteria; heterotrophic bacteria; manganese removal; acid resistance). The clustering results demonstrate the main research directions in the field of bacterial Mn(II) oxidation from 2008 to 2023.

## 4. Discussion

### 4.1. Research Hotspots and Trends

**Figure 9:**
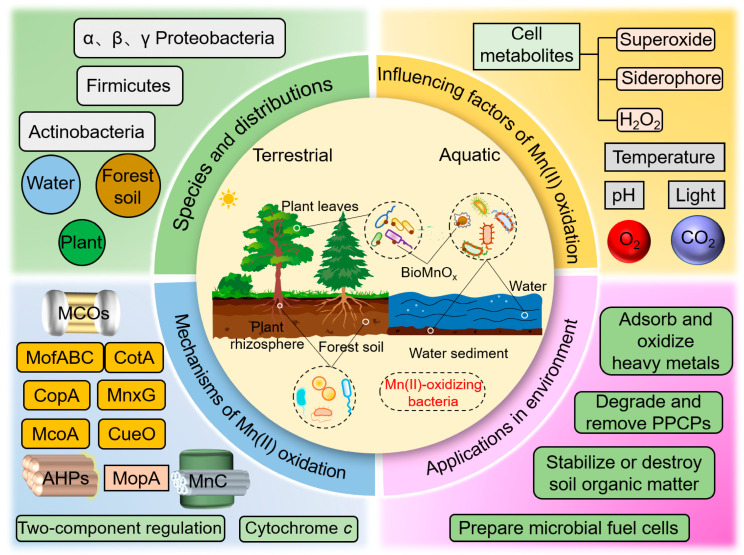
The summarized research hotspots of bacterial Mn(II) oxidation.

In this study, based on the combination of visualized knowledge graphs, burst analysis, and keyword clustering, we summarized and plotted the research hotspots, trends, and frontiers in this field in Figure 9, which are mainly distributed in the following aspects: species and ecological distribution; factors affecting the oxidation of bacterial Mn(II); the oxidation mechanism of Mn(II) in bacteria; and environmental applications.

#### 4.1.1. Species and Ecological Distribution

The keyword clustering involved in this section includes #6 manganese oxidizing bacteria, and the included keywords and keyword bursts include microbial community, moving bed biofilm reactor, Mn(II)-oxidizing bacteria, marine *bacillus*, strain SG-1, *Escherichia coli*, wastewater, groundwater, and heterotrophic bacteria. Specifically, the Mn(II)-oxidizing bacteria classification includes species such as *Proteobacteria*, *Actinobacteria*, and *Firmicutes* (Figure 9). Notable strains exhibiting Mn(II) oxidation activity include *Bacillus* sp. SG-1 [16,46], *Pseudomonas putida* MnB1 and GB-1 [39], *Leptothrix discophora* SS-1 [29], *Citreicella manganoxidans* sp. VSW210T [61], and *Celeribacter manganoxidans* DY25T [62]. In recent years, an increasing number of Mn(II) oxidizing co-cultured microbial systems have been discovered, where two microorganisms do not produce Mn(II) oxidation activity when cultured alone, but can produce Mn(II) oxidation activity when mixed, for example, *Arthrobacter* sp. and *Sphingopyxis* sp. [21], *Rhodopseudomonas palustris* and *Geobacter metallireducens* [63], ‘C*andidatus* Manganitrophus noduliformes’ and *Ramlibacter lithotrophicus* [64]. These Mn(II)-oxidizing bacteria are widely distributed in various environments, including forest soils, desert rocky desertification layers, springs, lakes, ponds, estuaries, caves, hydrothermal environments, contaminated sediments, seawater, seabed, drinking water, rhizosphere, and laboratory cultivation systems [57,65,66,67,68,69].

#### 4.1.2. Factors Influencing Bacterial Mn(II) Oxidation

The pertinent keyword clustering in this context includes #1 oxidative stress. The associated keywords and keyword bursts include oxidative stress, hydrogen peroxide, heme peroxidase, manganese-containing catalase, bioremediation, and superoxide dismutase. The bacterial Mn(II) oxidation process is exceedingly intricate and susceptible to a multitude of influencing factors, with substantial variations in the mechanisms employed by different Mn(II)-oxidizing bacteria. These factors primarily include external environmental variables, such as temperature, pH, oxygen concentration, carbon dioxide concentration, light exposure, extracellular polysaccharides, and metabolic byproducts generated by the bacteria (Figure 9) [15,63,70,71,72,73]. Moreover, other factors such as initial Mn(II) concentration, bacterial inoculum size, incubation time, metal ions, and other chemical reagents [26,55,72,74,75] can also significantly influence the bacterial efficiency in oxidizing Mn(II). Overall, bacterial Mn(II) oxidation is a complex process regulated by multiple factors, subject to the influence of external environmental conditions and endogenous metabolic byproducts.

#### 4.1.3. Mechanisms of Mn(II) Oxidation in Bacteria

The keyword clustering involved in this section includes #2 multicopper oxidase and #5 electron donors. The included keywords and keyword bursts are multicopper oxidase, manganese oxidation, biogenic Mn oxides, iron, spore, protein, expression, identification, mechanism, diversity, nitrification, and autotrophic denitrification. The mechanisms of Mn(II) oxidation in bacteria are mainly divided into direct and indirect oxidation. Direct oxidation refers to the direct oxidation of Mn(II) to Mn(III/IV) by Mn oxidases in bacteria through enzymatic reactions (Figure 9) [56,76,77,78]. The oxidation of Mn(II) is typically associated with the electron transfer chain, which transfers electrons through electron transfer proteins on the cell membrane to oxidize Mn(II) [79]. Indirect oxidation refers to using intermediate or secondary metabolites, such as superoxide radical and hydrogen peroxide, by bacteria to oxidize Mn(II) [26,72]. In addition, it has been recently discovered that ‘C*andidatus* Manganitrophus noduliformes and *Ramlibacter lithotropicus* rely on the oxidation of MnCO_3_ to support bacterial growth and proliferation in a co-culture environment, and the relationship between extracellular Mn(II) oxidation under aerobic conditions and autotrophic CO_2_ fixation in metabolic pathways has been further revealed through transcriptome analysis [64]. Comparative genomic analyses reveal that “*Ca*. Manganitrophaceae” share a core set of candidate genes for the Mn(II)-dependent chemolithoautotrophic lifestyle [80]. Of course, the specific Mn(II) oxidation mechanism may vary depending on the type and habitat of bacteria.

#### 4.1.4. Environmental Applications

The keyword clustering involved in this section includes #3 biofilter, #4 hexagonal birnessite, and #0 biogenic manganese oxide. The included keywords and keyword bursts are adsorption, activated carbon, manganese removal, drinking water, groundwater, biogenic manganese oxides, microbial community structure, pH, genetic diversity, bacterial hexagonal birnessite, birnessite, aerobic granular sludge, sediment, biodegradation, removal, transformation, kinetics, in situ, heavy metal, and aqueous solution Specifically, the BioMnO_x_ generated by Mn(II)-oxidizing bacteria can be utilized to remove and oxidize various heavy metals, organic pollutants, antibiotics, and so on (Figure 9). Mn(II)-oxidizing bacteria and their BioMnO_x_ efficiently removes heavy metals such as Pb, Cr, Cd, Cu, Fe, Ni, Pb, and Zn [52,81,82,83,84]. BioMnO_x_ can also be applied for the oxidation and removal of complex organic compounds and pollutants in pharmaceuticals and personal care products, such as textile dyes, antibiotics, painkillers, endocrine disruptors, biopesticides, and so on (Figure 9) [53,85,86]. In a word, Mn(II)-oxidizing bacteria and the produced BioMnO_x_ have extensive application prospects, including wastewater treatment, heavy metal removal, organic pollutant degradation, soil carbon sequestration, and battery material preparation, which are of great significance for environmental restoration and ecosystem elemental cycling.

### 4.2. Outlook

Bacterial-mediated Mn(II) oxidation has profound implications for global ecological balance and environmental changes. Researchers have previously screened and identified numerous Mn(II)-oxidizing bacteria from diverse environments; they have also cloned and verified various genes and oxidases associated with Mn(II) oxidation and characterized the produced BioMnO_x_ within bacterial cells. However, the process of bacterial Mn(II) oxidation remains highly intricate, and there are still many research directions worth further exploration and investigation, as follows:

(1) Isolating and screening Mn(II)-oxidizing bacteria from more diverse habitats. Mn(II)-oxidizing strains are predominantly sourced from aquatic ecosystems, such as marine and freshwater environments. Some research also focuses on soils and plants in mining areas. Exploring more Mn(II)-oxidizing bacteria from terrestrial habitats, extreme environments, or internal biological environments, such as plant leaves and rhizosphere and animal or insect intestines, is imperative. In addition, it is necessary to explore more co-cultured Mn(II) oxidation systems among microbial communities, especially chemolithoautotrophic bacteria. This interspecific cooperation and collaboration between dual or multiple bacteria from different sources can reveal more specific Mn cycling processes. The isolation and identification of these bacteria contribute to revealing the role of Mn(II)-oxidizing bacteria across the entire ecosystem;
(2) Utilizing multi-omics approaches to elucidate the Mn(II) oxidation mechanism and using genomics, transcriptomics, proteomics, metabolomics, and other technologies to collaboratively uncover novel genes involved in the Mn(II) oxidation mechanism. Then, exploring the regulation of Mn(II) oxidation-related metabolic networks by investigating the interactions among different genes;
(3) In situ monitoring of the process of Mn(II)-oxidizing bacteria producing BioMnO_x_. Through in situ monitoring, dynamic environmental monitoring data, and meteorological data, bacterial growth status and redox potentials can be obtained, thereby exploring the interactions between different compounds (nutrients, heavy metals, organic compounds, antibiotics, etc.) and Mn compounds in a regional environment and explaining the transformation rule of Mn valence state during the formation of BioMnO_x_. Such monitoring contributes to further understanding the role of Mn(II)-oxidizing bacteria in the ecological balance and biogeochemical cycling under global environmental changes;
(4) Transforming Mn(II)-oxidizing engineering bacteria to increase their applicability. Using synthetic biology methods, Mn(II) oxidation-related genes from different bacterial strains are fused, co-expressed, and modified to create novel and efficient Mn(II)-oxidizing engineering bacteria more suitable for rapidly degrading multiple substrates in complex environments. Subsequently, these engineered bacteria can be applied in producing bio Mn-based biological batteries, biomineralization, and metallurgy fields and are expected to play a significant role in developing renewable energy, mineral recovery, and environmental remediation.

## 5. Conclusions

Over the past fifteen years, the number of research papers published on bacterial Mn(II) oxidation has continuously grown. China has published the most research papers in this field, followed by the USA and Japan. The top three institutions that published the highest number of research papers are the Chinese Academy of Sciences, Oregon Health and Science University, and Harbin Institute of Technology. Bradley Tebo, Yaohui Bai, Jiuhui Qu, and Jie Zhang have published the most relevant research papers. At the same time, Bradley Tebo, Christopher Francis, Christopher Francis, and Deric Learman have received the highest number of citations. The published papers mainly focus on three disciplinary categories, namely “Environmental Science & Ecology”, “Chemistry”, and “Biotechnology & Applied Microbiology”, which align with the frequently cited journals in the field. The research hotspots in bacterial Mn(II) oxidation mainly focus on the species and distributions of Mn(II)-oxidizing bacteria, the influencing factors of Mn(II) oxidation, the mechanisms of Mn(II) oxidation, and their practical applications in environment. In conclusion, this study summarizes the current research status of bacterial Mn(II) oxidation from 2008 to 2023, tracks research hotspots, and explores future development trends, providing valuable insights for subsequent studies on the remediation of Mn(II) and other pollutants by Mn(II)-oxidizing bacteria.

## Figures and Tables

**Table 1:** Top ten countries by the number and centrality of publications.

Rank	Country	Count	Rank	Country	Centrality
1	China	197	1	USA	0.38
2	USA	128	2	China	0.34
3	Japan	33	3	Germany	0.15
4	Germany	20	4	Netherlands	0.14
5	India	18	5	England	0.09
6	Canada	11	6	Japan	0.04
7	England	10	7	South Korea	0.04
8	France	9	8	Pakistan	0.04
9	Australia	8	9	Mexico	0.04
10	South Korea	9	10	France	0.03

**Table 2:** Top ten institutions by the number of publications, centrality and their countries.

Rank	Count	Centrality	Institution	Country
1	41	0.19	Chinese Academy of Science	China
2	28	0.06	Harbin Institute of Technology	China
3	27	0.23	Oregon Health and Science University	USA
4	20	0.07	Huazhong Agricultural University	China
5	14	0.00	University of Chinese Academy of Sciences	China
6	12	0.00	Xi’an University of Architecture and Technology	China
7	9	0.05	Hiroshima University	Japan
8	8	0.04	Woods Hole Oceanographic Institution	USA
9	8	0.03	Smithsonian Institution	USA
10	8	0.00	Beijing University of Technology	China

**Table 3:** Top ten categories by the frequency and centrality of publications.

Rank	Category	Count	Rank	Category	Centrality
1	Environmental Sciences & Ecology	151	1	Environmental Sciences & Ecology	0.41
2	Environmental Sciences	122	2	Chemistry	0.41
3	Engineering	87	3	Biotechnology & Applied Microbiology	0.39
4	Microbiology	73	4	Biochemistry & Molecular Biology	0.34
5	Engineering, Environmental	72	5	Environmental Sciences	0.32
6	Biotechnology & Applied Microbiology	52	6	Engineering	0.18
7	Water Resources	39	7	Chemistry	0.18
8	Geology	32	8	Microbiology	0.15
9	Geosciences	28	9	Agriculture	0.14
10	Biochemistry & Molecular Biology	25	10	Toxicology	0.09

**Table 4:** Top ten authors and co-cited authors by the number of publications and citations.

Rank	Top Ten Productive Author	Count	Rank	Top Ten Co-Cited Author	Citation
1	Tebo BM	22	1	Tebo BM	212
2	Bai YH	13	2	Francis CA	104
3	Qu JH	12	3	Learman DR	94
4	Zhang J	10	4	Villalobos M	89
5	Pan XL	9	5	Geszvain K	87
6	Hansel CM	8	6	Dick GJ	80
7	Liu F	8	7	Webb SM	79
8	He ZF	7	8	Miyata N	74
9	Santelli CM	7	9	Anderson CR	71
10	Wei Z	7	10	Brouwers GJ	69

**Table 5:** Top ten cited journals.

Rank	Citation	Cited Journal	IF_2022–2023_	JCR	Country
1	301	*Applied and Environmental Microbiology*	4.32	Q2	USA
2	232	*Environmental Science & Technology*	11.09	Q1	USA
3	223	*Water Research*	12.75	Q1	England
4	214	*Geochimica et Cosmochimica Acta*	4.97	Q1	USA
5	205	*Geomicrobiology Journal*	2.30	Q3	USA
6	188	*Journal of Bacteriology*	3.06	Q3	USA
7	180	*Proceedings of the National Academy of Science of the United States of America*	10.71	Q1	USA
8	168	*Annual Review of Earth and Planetary Sciences*	14.29	Q1	USA
9	158	*Chemosphere*	8.80	Q1	England
10	153	*PLoS One*	3.64	Q2	USA

**Table 6:** Top ten co-cited references.

Title	Authors	Year	Citation Frequency
Synergistic effects of biogenic manganese oxide and Mn(II)-oxidizing bacterium *Pseudomonas putida* strain MnB1 on the degradation of 17 α-ethinylestradiol	Tran TN et al. [53]	2018	30
A novel manganese oxidizing bacterium-*Aeromonas hydrophila* strain DS02: Mn(II) oxidization and biogenic Mn oxides generation	Zhang Y et al. [55]	2019	29
Elimination of Manganese(II,III) Oxidation in *Pseudomonas Putida* GB-1 by a Double Knockout of Two Putative Multicopper Oxidase Genes	Geszvain K et al. [34]	2013	27
Mn(II, III) oxidation and MnO_2_ mineralization by an expressed bacterial multicopper oxidase	Butterfield CN et al. [56]	2013	23
Diverse manganese(II)-oxidizing bacteria are prevalent in drinking water systems	Marcus DN et al. [57]	2017	22
Effective start-up biofiltration method for Fe, Mn, and ammonia removal and bacterial community analysis	Cai YN et al. [52]	2015	22
Extracellular haem peroxidases mediate Mn(II) oxidation in a marine *Roseobacter* bacterium via superoxide production	Andeer PF et al. [26]	2015	21
CotA, a multicopper oxidase from *Bacillus pumilus* WH4, exhibits manganese-oxidase activity	Su JM et al. [51]	2013	21
Formation of manganese oxides by bacterially generated superoxide	Learman DR et al. [27]	2011	20
Identification of a third Mn(II) oxidase enzyme in *Pseudomonas putida* GB-1	Geszvain K et al. [39]	2016	20

**Table 7:** Top 15 keywords according to the frequency and centrality.

Rank	Keyword	Frequency	Centrality
1	Mn(II) oxidation	104	0.08
2	oxidation	92	0.16
3	iron	81	0.19
4	identification	65	0.10
5	removal	62	0.09
6	multicopper oxidase	61	0.04
7	water	42	0.06
8	oxides	42	0.07
9	spores	38	0.07
10	mechanisms	38	0.06
11	manganese oxidation	38	0.05
12	biogenic manganese oxides	34	0.09
13	microbial community	32	0.11
14	bacteria	32	0.08
15	adsorption	32	0.13

## Data Availability

All relevant data are within the manuscript and available from the authors upon request.
